# Biomechanical effects of exercise fatigue on the lower limbs of men during the forward lunge

**DOI:** 10.3389/fphys.2023.1182833

**Published:** 2023-08-17

**Authors:** Lidong Gao, Jingyi Ye, Kovács Bálint, Zsolt Radak, Zhuqing Mao, Yaodong Gu

**Affiliations:** ^1^ Faculty of Sports Science, Ningbo University, Ningbo, China; ^2^ Research Institute of Sport Science, University of Physical Education, Budapest, Hungary; ^3^ Research Academy of Medicine Combining Sports, Ningbo No 2 Hospital, Ningbo, China

**Keywords:** forward lunge, kinetics, kinematics, fatigue, lower limb

## Abstract

**Background:** During competition and training, exercises involving the lungs may occur throughout the sport, and fatigue is a major injury risk factor in sport, before and after fatigue studies of changes in the lungs are relatively sparse. This study is to investigate into how fatigue affects the lower limb’s biomechanics during a forward lunge.

**Methods:** 15 healthy young men participate in this study before and after to exposed to a fatigue protocol then we tested the forward lunge to obtain kinematic, kinetic changing during the task, and to estimate the corresponding muscles’ strength changes in the hip, knee, and ankle joints. The measurement data before and after the fatigue protocol were compared with paired samples t-test.

**Results:** In the sagittal and horizontal planes of the hip and knee joints, in both, the peak angles and joint range of motion (ROM) increased, whereas the moments in the sagittal plane of the knee joint smaller. The ankle joint’s maximum angle smaller after fatigue. Peak vertical ground reaction force (vGRF) and peak contact both significantly smaller after completing the fatigue protocol and the quadriceps mean and maximum muscular strength significantly increased.

**Conclusion:** After completing a fatigue protocol during lunge the hip, knee, and ankle joints become less stable in both sagittal and horizontal planes, hip and knee range of motion becomes greater. The quadriceps muscles are more susceptible to fatigue and reduced muscle force. Trainers should focus more on the thigh muscle groups.

## 1 Introduction

Lunge is an important part of a lower-limb muscle strength training and rehabilitation programs. This task is typically performed to enhance the lower limb muscles'-ability to generate higher force, especially in the quadriceps, and to minimize the risk of joint injury (Keogh, 1999), and develop functional postural balance (Ebben et al., 2009). Lunge requires more balance than the more commonly used deep squat thus it can be used more safely to strengthen the biarticular quadriceps and hamstrings muscles, which are necessary for appropriate rehabilitation of gait and daily living activities (Wilson et al., 2008; Dregney et al., 2023). Also, this task often makes sure that the athlete’s lower body and trunk become strong, flexible, and stable enough to support safe and efficient augmentation training (Tippett and Voight, 1995).

While modest knee loads support the health of the joint cartilage (Griffin and Guilak, 2005), high-intensity loads or extended exercise might cause injuries in the knee (Ebben et al., 2009; Richmond et al., 2013). Repeated work exposure to bow steps may also raise the incidence of joint degenerations, especially in the knee joint (Amin et al., 2008). For instance, repetitive knee flexion during work duties can lead to excessive fatigue (Richmond et al., 2013). Furthermore, the risk of injury increases when the intensity of exercise increases and activation of certain muscles increases or even exceeds the activation of other muscles (Irish et al., 2010).

Muscle fatigue, which is characterized by decreased muscular efficiency and force production capacity after extended exposure to activity (Gandevia et al., 2006; Yamane et al., 2023), has repeatedly been proven to have a negative impact on performance (Pincivero et al., 2000). In female athletes doing single-leg landings, neuromuscular fatigue has been demonstrated to alter knee flexion, knee abduction, and hip internal rotation (McLean and Samorezov, 2009). Moreover, the muscles’ diminished capacity for force production may affect their capacity to function as dynamic joint dampers (Pincivero et al., 2000) and may contribute to damage (Du and Fan, 2020). For instance, epidemiological research shows that greater incidence of badminton injuries are discovered during trainings because of muscle strength decreased (Shariff et al., 2009; Kondrič et al., 2011). In badminton, lunge is frequently employed as a fundamental step, and when players grow tired, they are more likely to suffer ankle injuries in addition to knee injuries during practice or competition (Herbaut et al., 2018). Garcia et al. (2012), however, showed the lunge squat is an exercise that accurately represents the tiredness process and enhances knee stability by equally activating the quadriceps and hamstring complexes, and this co-activation Weir et al. (1998) observed to occur simultaneously with fatigue. The fatigue process reduces the contraction force and muscle activation time of the quadriceps, which triggers an increase in hamstring muscle activity during fatigue (Garcia et al., 2012). By preventing external joint rotation, the quadriceps, hamstrings, and gastrocnemius muscles stabilize the knee (Winby et al., 2009). On the other hand, fatigue fractures are a common injury that typically arise from running exercise. Consequently, a vital initial step in guiding injury prevention research and informing injury screening is understanding how fatigue affects movement patterns in standard musculoskeletal screening procedures (Weeks et al., 2015).

Some studies have illustrated lunge and squat EMG and kinematic changes under different loading conditions (Stastny et al., 2015a; Stastny et al., 2015b), but fewer studies have been conducted after prolonged exercise or even fatigue. The muscle performance during extended exercise is unknown, although experienced gym athletes show greater muscular strength and better joint coordination compared to novice, which helps to minimize redundancy in force production through joint coordination and employing muscle power effectively (Phomsoupha and Laffaye, 2015).

There is a lack of many studies focusing on how fatigue induce kinematic and kinetics changing in lunge. There is a lack of information on the kinematics, kinetics, and changes in the force generation of the relevant muscle groups in the lunge before and after fatigue, despite the kinematic performance of the lunge in various directions (Comfort et al., 2015; Goulette et al., 2021; Park et al., 2021) and of closed chain exercises of the lower limb after fatigue (Gribble et al., 2007; Weeks et al., 2015; Du and Fan, 2020). Therefore, the aim of this study was to investigate the effects of fatigue on lower limb biomechanics during the anterior lunge before and after completing a fatigue protocol. Fatigue is a major contributor to injury risk, and relevant objective data provide the required understanding for understanding the cause of injury and to help improve injury prevention programs.

## 2 Materials and methods

### 2.1 Participants

**FIGURE 1:**
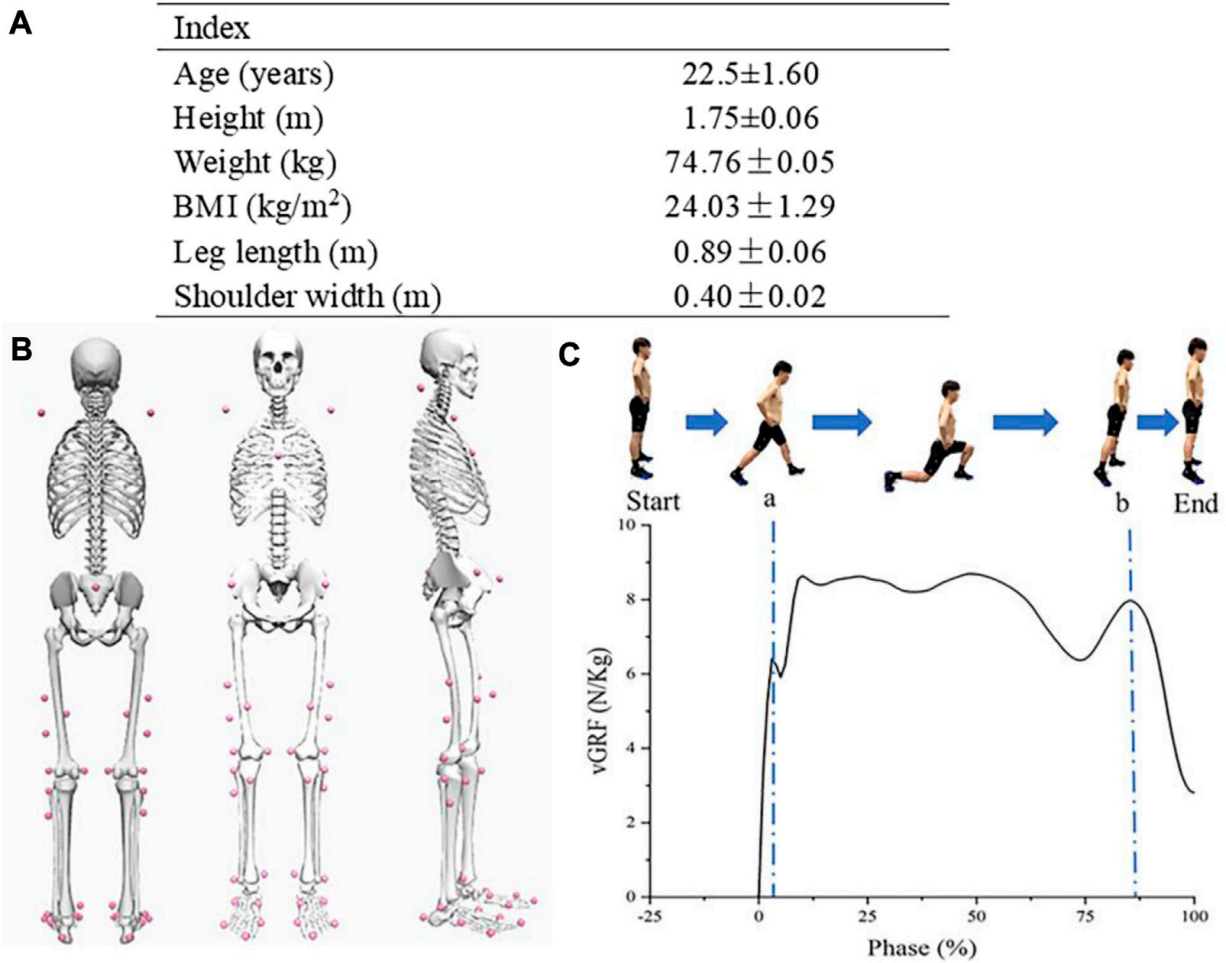
**(A)** Basic information of participants; **(B)** Back, front, and side view of the participant’s reflective markers; **(C)** Forward lunge process and corresponding vGRF; a, Initial impact peak; b, Drive-off peak.

The sample size was determined using data from previous studies (Nielsen et al., 2018; Liu et al., 2023). At least 15 participants were selected using G*Power3.1, Statistical test choosing Means: Wilcoxon signed-rank test (matched pairs) with tails of 2 and an alpha value of 0.05 and a power value of 0.80 and effect size of 0.80 (Faul et al., 2009). 15 young healthy males were recruited for this study, and relevant basic information is shown in Figure 1. The dominant leg was identified by self-declaration of the preferred leg during kicking which was defined as the dominant limb. Participants had no lower limb muscle or bone illness or injury 6 months prior to this testing session. Before the experiment, participants fasted for 2 h and were not allowed to drink any alcohol or caffeine for the following 24 h. To prevent shoes from influencing the experiment’s outcomes, they all wore shoes of the same design. All participants signed an informed permission form before the experiment and were made aware of the importance and goal of the study as well as the testing methods. The Ethics Committee of Ningbo University Research Institute approved the experiment. (NO: RAGH202303073005.2).

### 2.2 Instruments

An eight-camera Vicon motion capture system (Vicon Metrics Ltd., Oxford UK) was used to capture the markers motion trajectories with a sampling frequency of 200 Hz. Simultaneously with motion capture an embedded Kistler 3D force plate (Kistler, Switzerland) was used to record ground reaction forces with 1000 Hz. Before the experiment, 38 reflective markers (diameter: 14 mm) were mounted onto their bodies (Figure 1).

The EMG signals of the rectus femoris (RF), biceps longus (BF), tibialis anterior (TA), and gastrocnemius (GA) were recorded simultaneously at 1000 Hz with a Delsys EMG test system (Delsys, Boston, MA, and United States) to validate the results of the model. Maximum voluntary isometric contractions (MVIC) were performed for the rectus femoris, biceps femoris long head, tibialis anterior, and gastrocnemius muscles using a dynamometer (CON-TREX MJ System, CMV, Dübendorf, Switzerland).

### 2.3 Procedures

First, participants warmed up with 5 min run on a stationary motorized treadmill (h/p/cosmos sports and medical GmbH, Nussdorf-Traunstein, Germany). The leg length of each participant was measured manually with a metric ruler. We defined step length as the 70% of the leg length. The lunging leg position was marked with tape on the force platform for both side and starting position was also marked with tape with the corresponding distance for each participant leg length.

To obtain maximal values of EMG signals, participants performed a 5-s MVIC on a dynamometer, and each participant performed 3 consecutive measurements for each muscle group with 1-min rest interval. The MVIC was tested according to the recommended setting of the dynamometer, which allows participants to power up to the maximum force possible (Bliss and Dekerle, 2019). Professionals were on hand to guide and protect the participants throughout the test and secured the athlete’s trunk with a strap to avoid pronation. The participants were provided with concurrent visual feedback in the form of an isokinetic strength curve displayed on the dynamometer monitor. Verbal encouragement was also provided.

After skin preparation, shave off the body and clean the skin with alcohol. Bipolar surface electrodes were attached over four lower limb muscles. The sensor was placed over the muscle belly of the muscle parallel to the direction of the muscle fascicles. Bipolar surface electrodes (adhesive disposable electrode, 10 mm interelectrode distance) were taped parallel to muscle fascicles. Electrode placements was in accordance with the SENIAM recommendations (Hermens et al., 2000). The EMG signals of the RF, BF, TA, and GA muscles were collected at 1000 Hz using a wireless Delsys EMG test system.

Before the experiment, participants received specific instructions from an expert coach with several years of experience to make sure that participants were familiar with the task and that can perform the task properly. Proper executions had to meet the following criteria: 1) the body must remain upright and perpendicular to the horizontal at all times, with the arms crossed at the sides of the body; 2) the dominant leg’s thigh must be perpendicular to the ground; and 3) the non-dominant leg’s knee must be close to the ground but not touch it. Lunge started in a standing position with feet parallel to each other, at the previously marked starting location of the experiment. Then lunge was initiated by a verbal command. Based on the lunge forward of the dominant leg (right leg) to the mark on the force platform, participants were asked to moved their body weight downward to the upright leg and to maintain an upright posture. After the deepest position the non-dominant leg followed the dominant leg forward and settles parallel to the upright foot and stood on the corresponding ground marking, i.e., the body back into a standing position at the end. When a lunge is completed, the expert will indicate the quality of this movement, whether it reached the standard and how it can be improved. Unqualified movements are not included in the data.

Prior completing the fatigue protocol participants were familiarized with the fatigue protocol and instructed in the use of the Borg Scale RPE 6–20 scale (Borg, 1982), to obtain the level of the subjective fatigue. In addition, heart rate was also measured with a portable detector (Polar RS100, Polar Electro Oy, Woodbury, NY, and United States) to monitor the changes in heart rate (HR) changes during the fatigue protocol. In to imitate lower limb movement patterns that might be seen in a sporting or recreational situation (Weeks et al., 2015), fatigue protocol consisted 20 repetitions of forward lunges (alternating forward lunges with the dominant and non-dominant leg) and a 400-m of running at an individually maximal running speed on a motorized treadmill. If fatigue level was minimal after one set of fatigue protocols, then the following set of procedures were carried out, and so on. HR and RPE data are used to quantify fatigue (Du and Fan, 2020). When the participants were unable to continue the fatigue protocol due to exhaustion, or their heart rates reached 90% of the maximum heart rate (HRmax = 220-age) predicted for their age, or if their Borg scale scores reached RPE >17 (very difficult), fatigue was considered to be occurred. Participants were not informed about the number of repetitions to avoid intentional halting to save energy.

**FIGURE 2:**
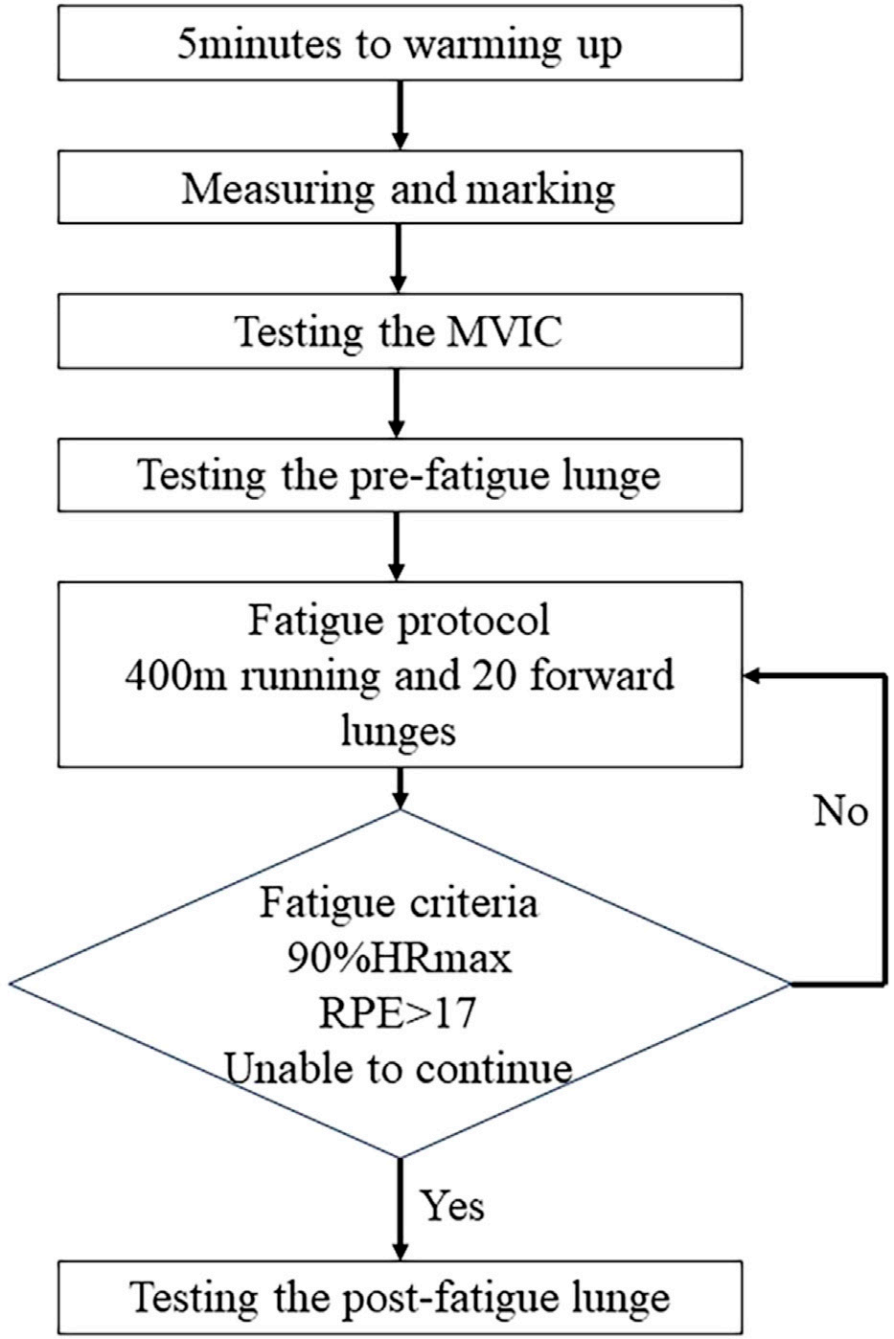
Flowchart of the study.

After the fatigue protocol was completed, we repeated the measured lung test. 5 min were given for placing reflective markers on to the participants body. For each person, six repetition was requested, three trial included in the analysis, the flow chart is shown in Figure 2.

### 2.4 Data collection and processing

One lunge data period was defined as starting from the previous frame of the dominant leg contacting the force table to the end of the next stance position. By collecting kinematic and kinetic data from the hip, knee, and ankle joints in the sagittal and horizontal planes, as well as vertical ground reaction forces (vGRF), joint changes under fatigue were examined. Lunge can be divided lunges into five phases recommended by Kuntze et al. (2010). In this study we used the first and last peak of the ground reaction force as initial impact peak and drive-off peak (Figure 1).

Joint moment, vGRF, and muscular strength data were normalized to body weight, respectively. Joint angular data, moment, and vGRF data were time-normalized to lunge strides (1–101 points), then these strides were averaged in a group level.

The original EMG signal is first pre-processed, including 50 Hz trapping to remove the industrial frequency interference, then 30 Hz zero-phase-shift high-pass filtering to remove motion artefacts; finally, the signal is taken to its absolute value, and the negative half-axis of the signal is flipped to the positive half-axis. Processing EMG signals in the 10–500 Hz frequency range using a bandpass fourth-order Butterworth filter (Zhang et al., 2010). The amplitude analysis is carried out using root mean square (RMS) calculation. RMS values of the EMG signals were calculated with a window size of 50 m. Using the same method for the EMG signal at MVIC, EMG values during lunge were normalized to the peak MVIC activity for each corresponding muscles. The MVIC and standardized activity value of each action was output. The EMG activity was calculated from 0 (0, completely inactive) to 1 (100%, fully activated) through the test root mean square amplitude/MVIC root mean square amplitude.

Marker trajectories and ground reaction force were filtered by zero-latency fourth-order Butterworth low-pass filters at 12 and 30 Hz, respectively. A threshold of 20 N was used, and ground reaction force data to determine the instant of the ground contact. The filtered marker coordinate data were imported into OpenSim for data processing after being converted in Matlab R2018a (The MathWorks, MA, United States) to formats recognized by OpenSim 4.3 (.mot and. trc) (Zhou et al., 2021). The model Mei et al. (2022) used in this experiment was adapted from the original OpenSim model (Rajagopal et al., 2016) by Mei et al. (2022). The model was upgraded with additional ranges of motion in the coronal and horizontal planes of the knee joint. First, the model was scaled to the static calibration measurement data using the positions and weights of the subjects’ marker points. Marker positions were manually corrected to reduce RMS error value (less than 0.02) between the experimental and virtual markers in the model. In order to reduce the error between the experimental and virtual markers, the joint angles were estimated using the inverse kinematics (IK) calculation tool in OpenSim. The findings were then optimized using least squares. The net moments of the hip, knee, and ankle joints are determined calculating inverse dynamics (ID) with a built in OpenSim algorithm. In order to determine the net forces and torques for each joint that creates the motion, the ID tool solves the force and acceleration mathematical equations of classical mechanics in an inverse dynamics sense (Yamane et al., 2023). The next step involved estimating muscle forces and muscle activation using static optimization (SO) and minimization of the sum of squared muscle activity. The Delsys EMG test system measurements of standardized muscle electrical signals were used to validate the model, which was then compared to the OpenSim static optimization algorithm’s output of the degree of muscle activation. The quadriceps, hamstrings, gastrocnemius, and tibialis anterior muscles were among the major muscle groups whose forces were estimated using static optimization.

### 2.5 Statistical analysis

Data are presented as the Mean Difference and Standard Deviations (MD±SD). To check the normality of each dataset we used Shapiro-Wilk normality test. Paired samples t-tests were used to compare joint ROM, peak joint angles, peak joint moments, peak vGRF, and two peaks (A and B) of the lunge test before and after the fatigue protocol. The direction of the movement was also marked as follows: 1; flexion (+)-extension (−) in the sagittal plane and internal rotation (+)-external rotation (−) in the horizontal plane, 2; knee joint was flexion (+)-extension (−) in the sagittal plane and internal rotation (+)-external rotation (−) in the horizontal plane. 3; ankle sagittal plane was dorsiflexion (+) and plantarflexion (−). IBM SPSS Statistics 26 (IBM Corporation, Armonk, NY) was used to calculate all statistical analysis. The alpha level was set at 5%.

## 3 Results

### 3.1 Hip, knee, and ankle joint angles

As can be seen in Table 1, there was a significant difference in the hip’s maximum angle both in the sagittal plane and horizontal plane before and after fatigue (*p* = 0.007 and *p* = 0.017), representing an increase of 4.38° and 3.35°. And hip minimum peak joint angle was significantly lower (*p* = 0.011) in the horizontal plane after fatigue. There was a 5.75° increase in ROM and a significant difference in maximal and ROM at the knee joint (both *p* = 0.001) in the sagittal plane. And for the knee in the horizontal plane after fatigue, the maximum value has, increased by 11.99°, a significantly higher (*p* < 0.001), ROM also differed significantly before and after fatigue (*p* = 0.001), with an increase of 10.32°. The maximum and minimum values in the ankle were significantly different (both *p* < 0.001). The ROM was nearly unchanged, while the maximum value decreased by 7.49° and the minimum value dropped by 7.74°.

**TABLE 1:** Hip, knee and ankle range of motion and maximum and minimum peak angles in the Sagittal plane and Horizontal plane before and after fatigue protocol.

Index		Pre		Post	
		S	H	S	H
Hip	Max	88.51 ± 12.36*	9.97 ± 6.54*	92.89 ± 9.23*	13.32 ± 2.56*
	Min	12.87 ± 11.72	0.46 ± 5.79*	16.80 ± 5.21	1.58 ± 2.09*
	Rom	75.64 ± 5.74	10.63 ± 2.76	76.10 ± 9.53	11.75 ± 3.38
Knee	Max	−120.45 ± 10.45*	13.44 ± 11.46*	−124.44 ± 8.34*	25.43 ± 5.07*
	Min	−20.90 ± 8.21	−12.15 ± 8.42	−19.14 ± 7.11	−10.48 ± 5.15
	Rom	99.55 ± 7.71*	25.59 ± 12.08*	105.30 ± 8.27*	35.91 ± 3.92*
Ankle	Max	32.05 ± 8.40*	—	24.56 ± 4.55*	—
	Min	−0.48 ± 5.83*	—	−8.22 ± 3.40*	—
	Rom	32.53 ± 4.40	—	32.78 ± 5.73	—

* indicates significant difference between pre and post, *p* < 0.05. pre, pre-fatigue; post, post-fatigue. S, sagittal plane; H, horizontal plane. (°).

### 3.2 Hip, hip, and ankle joint moments

As shown in Table 2, there was a significant difference in the peak sagittal plane moment at the hip joint before and after fatigue (*p* < 0.001), with an increase in moment of 0.39 Nm/kg. There was also a significant increase in the horizontal plane (*p* = 0.003) as well. The moment at the knee joint significantly decreased in the sagittal plane by 0.24 Nm/kg (*p* = 0.004). At the ankle joint, the sagittal plane moment was statistically significant after fatigue (*p* = 0.023). At the ankle joint, the post-fatigue sagittal moment was statistically significant (*p* = 0.023).

**TABLE 2:** Peak joint moments in the sagittal and horizontal plane before and after fatigue protocol.

Index		Pre	Post
Hip	S	−1.95 ± 0.53*	−2.34 ± 0.17*
	H	0.38 ± 0.10*	0.48 ± 0.08*
Knee	S	1.54 ± 0.68*	1.30 ± 0.30*
	H	−10.98 ± 1.38	−11.48 ± 2.15
Ankle	S	−1.12 ± 0.13*	−1.06 ± 0.14*

* indicates significant difference between pre and post, *p*< 0.05. pre, pre-fatigue; post, post-fatigue. S, sagittal plane; H, Horizontal plane. (Nm/kg).

### 3.3 Hip, hip, and ankle joint moments

Table 3 shows that only the initial contact peak (A) for vGRF before and after fatigue had a statistically significant decline of 0.49 N/kg (*p* = 0.014). The mean peak and the drive-off peak (B) were not statistically significant, but the mean peak increases while the drive-off peak decreases.

**TABLE 3:** Peak vGRF, of the first peak and last peak before and after fatigue protocol.

Index	Pre	Post
vGRF	9.77 ± 0.38	9.26 ± 1.95
A	7.15 ± 0.80*	6.66 ± 0.59*
B	8.39 ± 1.22	8.51 ± 1.13

* indicates significant difference between pre and post, *p*< 0.05; A, initial impact peak; B, Drive-off peak. pre, pre-fatigue; post, post-fatigue. (N/kg).

### 3.4 Model validation and muscle force

**FIGURE 3:**
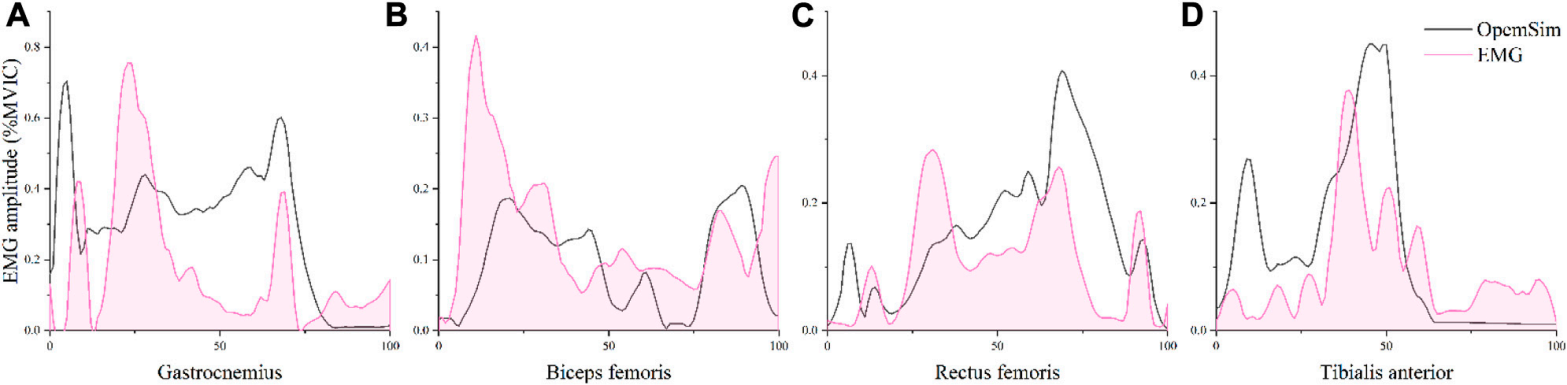
The comparison of gastrocnemius **(A)**, biceps femoris **(B)**, rectus femoris **(C)**, and tibialis anterior **(D)** EMG activity levels obtained using the EMG signal and the estimated values from the OpenSim optimization algorithm.

**FIGURE 4:**
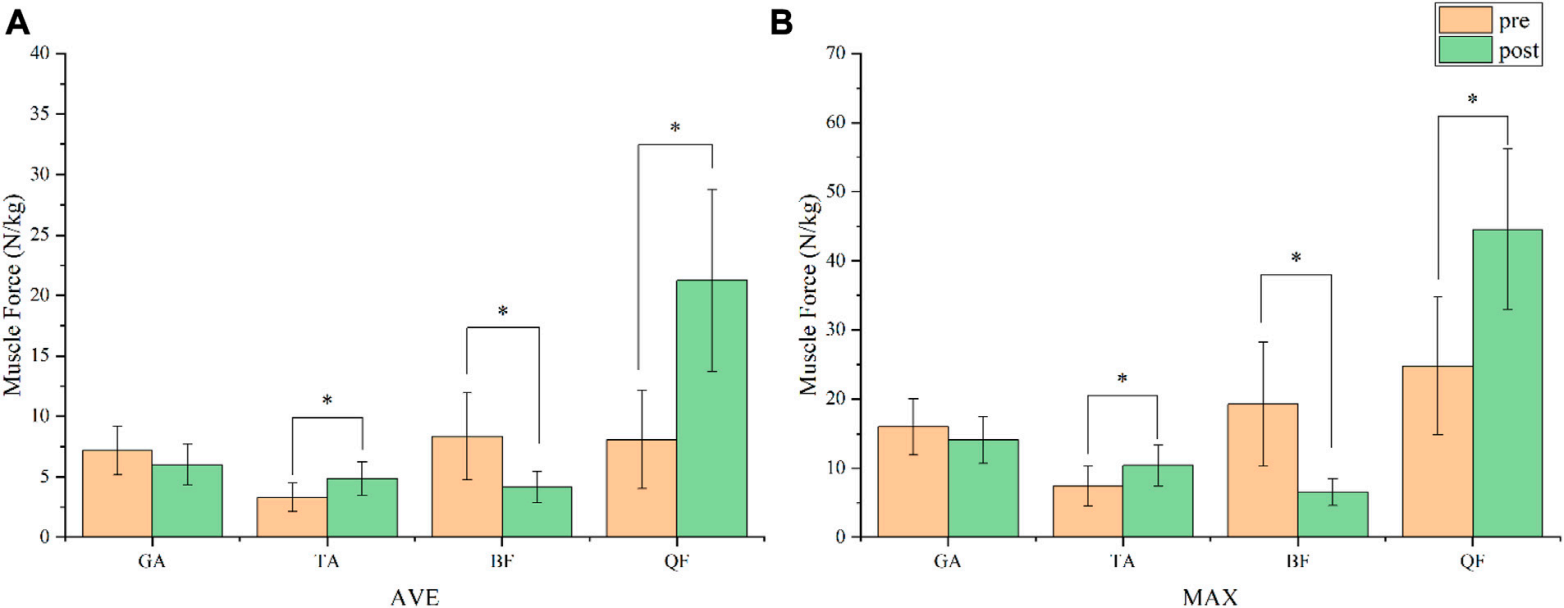
Comparison of the average **(A)** and maximal **(B)** estimated muscle force during lunge before and after fatigue protocol. GA, gastrocnemius; TA, tibialis anterior; BF, biceps femoris; QF, quadriceps femoris. *, *p* < 0.05.

According to Figure 3, muscle activity during lunge using the OpenSim SO tool matched the experimentally obtained EMG activity signal trends, proving that the data from the OpenSim model in this study is reliable (Hamner and Delp, 2013). When compared to muscle forces before fatigue (Figure 4), the biceps femoris and gastrocnemius both had lower average and maximal muscle force after fatigue protocol. The quadriceps and tibialis anterior muscles generated more force on average and at their maximal force output was also higher. The average muscle force of the tibialis anterior muscle was increased by 1.56 N/kg compared to after fatigue, it has a significant difference (*p* = 0.001); its maximum muscle force increased by 3.00 N/kg, also a significant difference, (*p* = 0.046). The mean muscle force of the biceps femoris decreased by 4.19 N/kg (*p* < 0.001). The maximum muscle force was significantly different, decreasing by 12.76 N/kg (*p* = 0.005). For the quadriceps, mean muscle force significantly increased by 13.16 N/kg (*p* = 0.002), and maximum muscle force increased by 19.78 N/kg (*p* < 0.001). Of the muscles explored, the quadriceps had the most significant increase in mean and maximum muscle force.

## 4 Discussion

The aim of this study is to investigate the changes of lunge biomechanics before and after completing a fatigue protocol in healthy young men. Our results provide the necessary insights by using our objective data to understand which aspects of the lower limb are specifically affected by fatigue, and help to prevent the injuries associated with fatigue based on previous research. Fatigue needs to be taken into account as part of a risk assessment for injuries where internal workload, cardiovascular fitness, and fatigue interact (Benjaminse et al., 2019; Song et al., 2023). Running and forward lunge a chosen as the fatigue protocol for this study because it more accurately simulates the fatigue brought on by performing exercise repeatedly.

In both the sagittal and horizontal planes of the hip joint, according to our research, the peak angle increased in the sagittal plane and horizontal plane. On both planes, ROM also increased differently. Peak angle and ROM in the sagittal plane both significantly increased in the knee. The action of the knee joint and the motion of the hip joint during lunging are very similar. Greater knee flexion and forward trunk movement as a result of tiredness can also be used to explain increased hip flexion (Lin et al., 2015). The upper trunk’s swing is enhanced by fatigue, and limb movements are necessarily less effective as maintaining the trunk becomes difficult. Similar findings were made by Weeks et al. (2015) who hypothesized that fatigue increases hip flexion, pelvic tilt, inclination, and rotation as well as trunk flexion and lateral rotation. As a result of fatigue, fatigue causes the trunk and pelvis ROM become greater. The position of the body’s center of mass and the subsequent loading of the lower limb joints may be dramatically altered by the trunk because of its greater relative mass (Park et al., 2021). The peak knee joint angle and ROM increased significantly after the fatigue protocol compared to pre-fatigue in the sagittal plane. This shows that when we are exhausted or when the intensity of the exercise builds up over time, the knee joint tends to move farther forward from a vertical ground position. According to Zellmer et al. (2019), increased knee flexion may also increase the knee’s tendency to move farther forward. And he also finds, peak knee joint stress, quadriceps force, knee moment, and knee flexion are higher when the knee is brought in front of the toes during the lunge. Less stretching force is placed on the patellar tendon when the knee is kept behind the toes (Escamilla et al., 2008). The plantar flexor and foot muscles involved more to take transfer the additional stress required to maintain the joint output when the hip and knee muscles are working less efficiently due to the fatigue effect. In the horizontal plane, the knee joint found a considerable shift as well, showing an increase in peak angle and ROM. This outcome, in our opinion, may be a result of greater toe external rotation movement in the upright foot during landing and more forward movement of the knee. Because fatigue has been shown to lead to reduced control of the stabilizing muscles in the hip and knee joints, resulting in altered joint kinematics (Webster et al., 2012). Different degrees of knee flexion may result in different levels of sports performance and joint impact forces. Athletes with knee injuries increase knee flexion during the lunge to reduce the impact of landing on the joint and increase their dynamic stability center of mass by reducing their knee height (Huang et al., 2014). And Huang et al. (2014) noted that to reduce the likelihood of damage, athletes may flex their knees more and conduct less physically demanding activities. This may be a neuromuscular protective mechanism. If athletes want to improve performance, adding greater weights is the most popular and important method of resistance training (Comfort et al., 2015). In addition, the increased external weight causes more mechanical work to be done at the hip and ankle joints (Riemann et al., 2012), muscle activation was significantly higher in the weight-bearing condition than in the self-weight condition.

While joint ROM did not vary significantly in the ankle joint, the peak angle did, with the maximum joint angle falling by almost 7.49° and the minimal angle increasing by 7.74°. This indicates that following fatigue, the ankle joint is more likely to be at a more plantarflexed position before landing. According to previous study, a considerable 10° increase in ankle plantarflexion increases the risk of injury owing to calf muscle fatigue and foot overuse and may also produce fatigue and injury to the Achilles tendon and anterior heel ligaments (Mei et al., 2017). Participants showed reduced dorsiflexion, possibly as a result of fatigue, leading to an insufficient dorsiflexion muscular force generation and relatively high plantarflexion muscle force. Additionally, the vGRF can also be used as a measure for resistance training, which can be used to improve performance, reduce injury risk, and increase strength (Dempster et al., 2021). According to our finding, peak vGRF falls down after fatigue, and peak contact reduces down to around 0.49 N/kg. A larger portion of the heel contact the ground and a better transmission of forces across the ankle muscles when the heel touches the ground are made possible by higher ankle plantarflexion, which can possibly reduce the risk of injury.

In terms of joint moment, the peak hip moment increased significantly in the sagittal plane and horizontal plane, while at the knee joint it decreased in the sagittal plane and increased in the horizontal plane. The ankle joint showed a significant reduction in the moment. According to previous research (Kovács et al., 2023), an increase in moment at the hip joint was linked to a change in kinematics, including an increase in flexion angle brought on by forward body lean. Fatigue reduces the activation of the vastus lateralis muscles during the lunge and the knee moment in the upright leg during the lunge. The quadriceps, hamstrings, and gastrocnemius are the primary tibiofemoral joint motor muscles. These muscle groups make up nearly all of the cross-sectional area of the knee muscular tissue, or 98% (Wilson et al., 2008). The co-activation of the quadriceps and hamstrings, as well as the activation of the biceps femoris and rectus femoris, were unaffected by fatigue. When becoming fatigued, lateral femoral muscle activation may decrease and hamstring co-activation may rise. This strategy may be intended to reduce knee flexion by increasing resistance to diagonal joint motion because co-activation between the lateral femoral and biceps femoris muscles has a tendency to increase after fatigue (Longpré et al., 2015). (Longpré et al., 2015) speculated that a significant decrease in knee moments may result from the vastus lateralis and biceps femoris muscles’ ability to cooperate more when they are subjected to fatigued. An increase in plantarflexion and a decrease in the dorsiflexor muscles’ force may be responsible for the drop in ankle net moments. Du’s reported Du and Fan (2020) that there was a tendency for ankle plantarflexion to increase after fatigue, although the foot landed more flat position (i.e., greater contact area with the ground) at the ground. The findings of this study also indicate that better-performing athletes had stronger muscle strength and displayed higher knee and ankle moments, suggesting that the results of his experiment may be related to the participants’ varying levels of activity.

The forward lunge is a great exercise to stimulate lower limb extensor muscles, including the hip and knee extensor muscles (Ross, 1997). The average and maximum muscle force of the biceps femoris and gastrocnemius muscles decreased based on the estimation of the OpenSim SO calculation. The quadriceps and tibialis anterior muscles experienced significant increases in both their average and maximal muscular forces. The quadriceps muscle showed the highest increase in maximum force increase by a 19.78 N/kg. This demonstrates that the quadriceps muscle gets the most work done and it is the most active muscle during forward lunge. Running fatigue throughout the fatigue protocol may have been the source of the decline in biceps femoris muscle strength. Moreover, the maximum muscle force of the biceps femoris decreased by 12.76 N/kg. In contrast, the increased force in the tibialis anterior muscle may be the result of the knee joint moving more forward after exhaustion. For the aim of inducing localized tiredness in the quadriceps and other lower limb muscles, the forward lunge in the fatigue protocol was used, and this is what the study explored. The results of this study help us to determine which of the investigated muscles that are heavily involved in the lunge movement during the post-fatigue and furthermore it can help us to design more specific training exercise for these muscles. According to Baker et al. (1998), a variety of exercises at a moderate speed should be performed than for a high resistance strength training can be started in order to gain additional muscle strength and muscle mass. Although high level of fatigue is believed to enhance these aspects of muscle function, thus increasing the amount of fatigue that working muscles experience may benefit in improving strength and hypertrophy (Rooney et al., 1994).

The front lunge has the advantages of increasing safety when it is properly executed, preventing potential knee injuries, and building strong postural muscles (Wilson et al., 2008). Certain forms of the lunge can also be added into the fitness regime if you want to improve your fitness. These movements can help to improve the dynamic flexibility of the lower limb joints and aiding in muscle hypertrophy and force development. Importantly, they can also be used to prevent injuries so that augmentation training can be carried out effectively and safely (Tippett and Voight, 1995). In general, it is important to perform all relevant exercises according to your condition to avoid unnecessary injuries caused by fatigue.

The methods we used in this study has limitation which needs to be addressed. First of all, although the biomechanical alterations in the forward lunge before and after fatigue protocol were investigated, they were not compared between different fatigue states. Secondly, The SO uses known movements of the model to solve for muscle activation forces, so the results may differ from the EMG test system, this means that there are limitations of the muscle force estimation we used. Thirdly, there is regional difference in muscle EMG activity and the location of measurements is not always the same for each participant, so EMG is also limited. Finally, we only tested the healthy young men, and we did not test other populations such as the athletic and injured populations, besides the protocol is limited.

## 5 Conclusion

In this study, we discovered that forward lung is affected by fatigue. After fatigue, the hip and knee joints have a greater ROM in the sagittal plane and an increased offset in the horizontal plane, with a tendency towards forward leaning. In addition to an increased amount of plantarflexion muscle activity in the ankle and unstable body postural performance. Additionally, the quadriceps muscles are the most affected after fatigue. To avoid injuries due to instability after fatigue, training people should focus more on strength training of the hip and knee muscles groups as a way to control joint motion more effectively.

## Data Availability

The raw data supporting the conclusion of this article will be made available by the authors, without undue reservation.
